# Supplementary material for: Effect of N-Acetylcysteine Administration on 30-Day Mortality in Critically Ill Patients with Septic Shock Caused by Carbapenem-Resistant Klebsiella pneumoniae and Acinetobacter baumannii: A Retrospective Case-Control Study
Source: Antibiotics (Basel). 2021 Mar 8;10(3):271. doi: 10.3390/antibiotics10030271 (PMC8001571; doi:10.3390/antibiotics10030271)

Supplementary Figure1. Treatment protocol in critically ill patients with suspected Gram-negative infection implemented by Infectious Diseases consultants at the Intensive Care Unit of IRCCS Neuromed (Pozzilli) and Sapienza University of Rome.

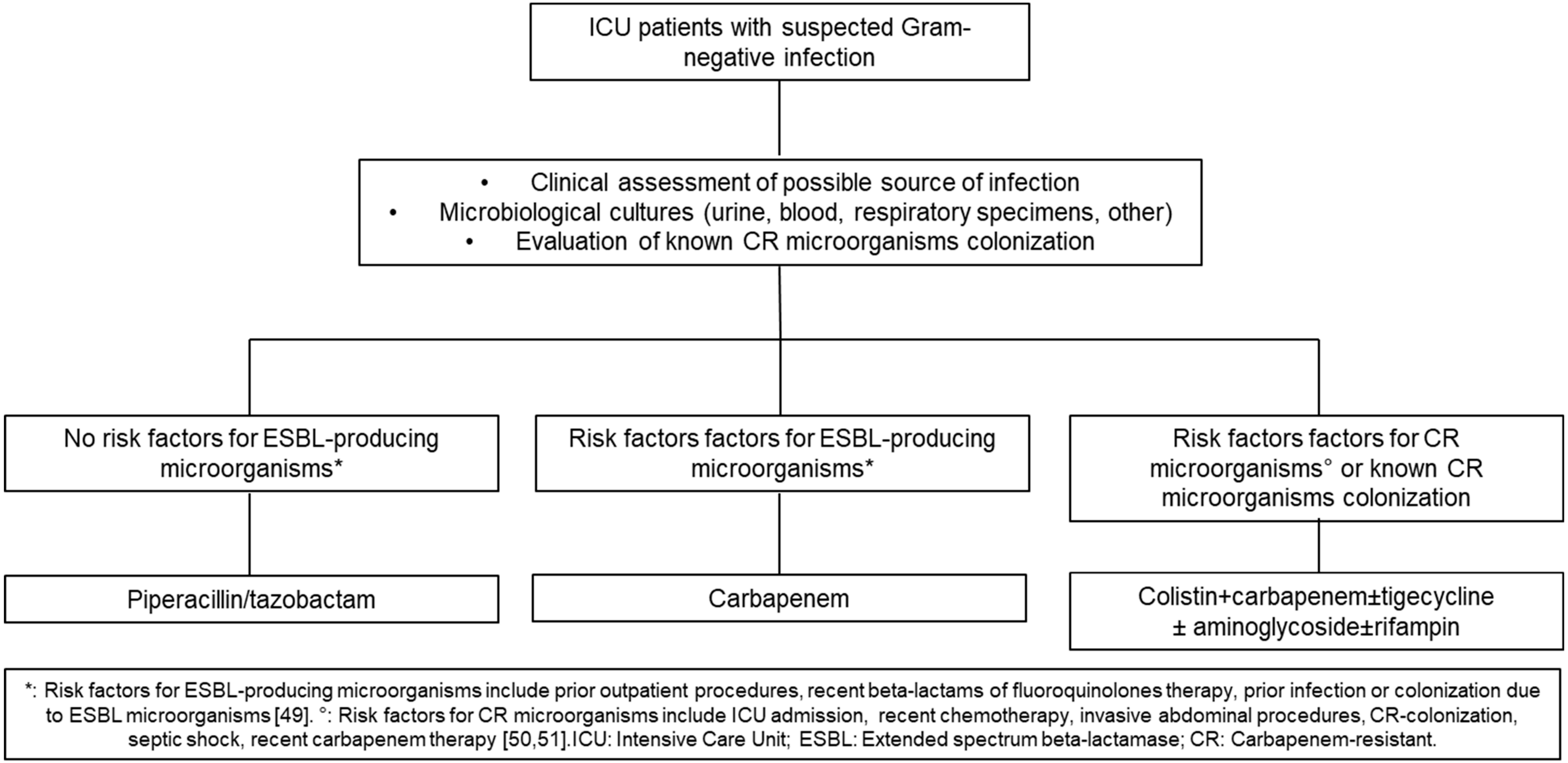

Supplement: Supplementary file 1 [file antibiotics-10-00271-s001.pdf]
